# Study protocol: the empirical investigation of methods to correct for measurement error in biobanks with dietary assessment

**DOI:** 10.1186/1471-2288-11-135

**Published:** 2011-10-05

**Authors:** Derrick A Bennett, Julian Little, Lindsey F Masson, Cosetta Minelli

**Affiliations:** 1 Clinical Trial Service Unit and Epidemiological Studies Unit (CTSU), University of Oxford, Old Road Campus. Roosevelt Drive, Oxford, OX3 7LF, UK; 2 Epidemiology and Community Medicine, University of Ottawa, 451 Smyth Road, Ottawa, Ontario, K1H 8M5, Canada; 3 School of Medicine and Dentistry, Division of Applied Health Sciences, University of Aberdeen, Polwarth Building, Foresterhill, Aberdeen, AB25 2ZD, UK; 4 Institute of Genetic Medicine, European Academy of Bolzano (EURAC), Viale Druso 1, 39100 Bolzano, Italy

## Abstract

**Background:**

The Public Population Project in Genomics (P^3^G) is an organisation that aims to promote collaboration between researchers in the field of population-based genomics. The main objectives of P^3^G are to encourage collaboration between researchers and biobankers, optimize study design, promote the harmonization of information use in biobanks, and facilitate transfer of knowledge between interested parties. The importance of calibration and harmonisation of methods for environmental exposure assessment to allow pooling of data across studies in the evaluation of gene-environment interactions has been recognised by P^3^G, which has set up a methodological group on calibration with the aim of; 1) reviewing the published methodological literature on measurement error correction methods with assumptions and methods of implementation; 2) reviewing the evidence available from published nutritional epidemiological studies that have used a calibration approach; 3) disseminating information in the form of a comparison chart on approaches to perform calibration studies and how to obtain correction factors in order to support research groups collaborating within the P^3^G network that are unfamiliar with the methods employed; 4) with application to the field of nutritional epidemiology, including gene-diet interactions, ultimately developing a inventory of the typical correction factors for various nutrients.

**Methods/Design:**

Systematic review of (a) the methodological literature on methods to correct for measurement error in epidemiological studies; and (b) studies that have been designed primarily to investigate the association between diet and disease and have also corrected for measurement error in dietary intake.

**Discussion:**

The conduct of a systematic review of the methodological literature on calibration will facilitate the evaluation of methods to correct for measurement error and the design of calibration studies for the prospective pooling of biobanks. This could increase the efficiency of the design of such studies, improve statistical power, reduce bias, and aid in the assessment of gene-environment interaction effects in complex diseases. The systematic review of calibration of dietary intake information could inform gene-diet interaction investigations involving the pooling of results from studies with nutritional data collected in different ways.

## Background

Large prospective cohort studies that have collected DNA source material (such as blood, saliva or other buccal specimens) with the aim of assessing both genetic and gene-environment (G × E) interaction effects on common complex diseases are becoming more widespread [1,2]. It is well known that the statistical power when testing for interactions is much lower than that when testing for main effects and thus require very large sample sizes [3-5]. A further complication of the assessment of G × E interactions is that they are usually subject to measurement error, which further reduces the statistical power to detect these types of interactions reliably. Errors in measurement of environmental exposure variables lead to biased estimates of effect sizes (e.g. relative risks or odd ratios). In the simplest case of measurement error in a single environmental exposure independent of disease status, the bias will be conservative [6]. If the environmental exposure measurement error is substantial, then even relatively small errors in genotyping within limits that are often quoted (around 3%) can have an appreciable effect on interaction estimates [7]. In more complex situations such as errors in confounders or differential errors in an environmental exposure between diseased cases and healthy subjects, the bias can operate positively or negatively [8]. The bias can also be in either direction when genotyping errors are differential. When G and E are independent in the source population, and the errors in the assessment of each are independent, both differential and non-differential misclassification of a dichotomous factor tends to underestimate departure from a multiplicative G - E joint effect. The impact of misclassification on departures from additive effects is difficult to predict [9,10].

In theory, case-control studies are more vulnerable to differential misclassification than are cohort studies (and the related case-cohort and nested case-control designs). However, provided that the extent of misclassification of exposure does not vary by genotype, differential misclassification between cases and controls may not be a serious problem for the detection of departures from a multiplicative G - E joint effect [9]. Simulation analyses under more general joint misclassification of genotype and environmental exposure, again focussing on detection of departure from multiplicative joint effects, also suggest that many regular tests for the hypothesis of no interaction maintain correct type I error rates in the presence of differential misclassification when there is no effect or a weak marginal effect of genotype [11]. Regardless of this fact, however, there will still be a loss of statistical power hence the need for large sample sizes.

If it is possible to quantify the magnitude of the measurement error, then making appropriate adjustments for it (i.e. calibration of the ensuing effect size), can add great value to the study of such environmental exposures, particularly in terms of preventing a reduction in statistical power. Calibration studies that involve a subset of participants from the main study can be designed in parallel with the main study in order to assess the degree of measurement error in the environmental exposures of interest, and produce calibration/correction coefficients [12-14]. In order to obtain sample sizes large enough to detect G × E interactions reliably, networks of researchers involved in such biobanks need to be formed in order to conduct collaborative projects across a variety of centres. This requires harmonisation across the centres in terms of methods used to measure exposure to a particular environmental factor of interest, which often vary from centre to centre. However, if the relationships between methods, as well as their error structures, were known, this problem could be addressed by use of correction and calibration coefficients, which would allow harmonisation of the results obtained from the different methods. Availability of a "comparison chart" of approaches to correct for measurement accompanied by an inventory of correction/calibration coefficients for methods used to measure an environmental exposure would represent a valuable resource for research on G × E interactions.

Dietary intake is a particularly challenging environmental exposure to measure, and studies of diet-disease associations (and gene-diet interactions) are limited by the difficulty in obtaining accurate estimates of long-term habitual intake. In addition, the different strengths and weaknesses of the various dietary assessment methods result in different degrees of accuracy in their estimates of habitual intake, which further complicates the pooling of data from multiple studies. Information from calibration studies can be used to a) provide design information [e.g. optimal sample size needed]; b) assess the relationship between different dietary assessment methods; and c) determine the degree of measurement error in estimates of association between diet and disease [15].

The Public Population Project in Genomics (P^3^G) is an organisation that aims to promote collaboration between researchers in the field of population-based genomics. The main objectives of P^3^G are to cultivate collaboration, optimize study design, set-up and research activities of population-based biobanks, devise schemes to promote harmonisation of biobanks, and facilitate transfer of knowledge between group members and other interested parties. P^3^G currently describes over 150 biobanks throughout its catalogues [16]. The importance of calibration and harmonisation of methods for environmental exposure assessment to allow pooling of data across studies in the evaluation of G × E interactions has been recognised by P^3^G, which has set up a methodological group on calibration with the aim of: 1) reviewing the published methodological literature on measurement error correction methods with assumptions and methods of implementation; 2) reviewing the evidence available from published nutritional epidemiological studies that have used calibration studies; 3) disseminating information in the form of a comparison chart on approaches to perform calibration studies and how to obtain correction factors in order to support research groups collaborating within the P^3^G network that are unfamiliar with the methods employed; 4) with application to the field of nutritional epidemiology, including gene-diet interactions, ultimately develop an inventory of the typical correction factors for various nutrients. In this project, we will systematically review the methodological literature on measurement error correction methods and the nutritional epidemiological literature on calibration studies of dietary assessment, a field of central importance in the study of G × E interactions and for which harmonisation is greatly needed.

## Methods/Design

### Study eligibility

For the methodological component of the review manuscripts among any population(s) throughout the world are potentially eligible for inclusion in this overview if they satisfy the following criteria: (1) they consider the issue of measurement error using some form of calibration study (i.e. reliability study [compared several instruments one of which may be a "gold-standard"] or validation study [a "gold-standard" compared with another measurement instrument], or reproducibility study [replicate measurements of a measurement instrument]); (2) the method was motivated by, and applied to, a real dataset (3) the statistical assumptions of the method are clearly described [e.g. can the method deal with differential as well as non-differential measurement error]; (4) the advantages of the method over other possible methods of correcting for measurement in terms of precision and validity are clearly described; (5) the most appropriate study design for implementation of the method is described; (6) details on whether the method can be implemented in standard statistical software are reported.

For the nutritional epidemiology component of the review manuscripts of studies conducted among any population(s) throughout the world are potentially eligible for inclusion in this overview if they satisfy the following criteria: (1) dietary intake was estimated with either a questionnaire, weighed records, non-weighed diet diary, 24-hour recall, or a biochemical marker; (2) the research team conducted some form of calibration study in order to correct for measurement error in estimation of dietary intake; (3) correction factors are reported for the dietary exposures measured.

### Study identification

Methodological studies will be identified from searches of statistical and mathematical databases [JSTOR, Current Index to Statistics, MathSciNet, and Scopus (also covers all of the health-related journal titles included in MEDLINE and EMBASE)]. Nutritional epidemiological studies will be identified by a range of methods including computer-aided literature searches of medical databases [MEDLINE, EMBASE, BIOSIS, CINAHL, CAB Abstracts (for Human Nutrition), Scopus, Dietary Assessment Calibration/Validation Register, and LILACS]. We will also scrutinise the reference lists of study reports and review articles, and by inquiring among collaborators and colleagues. Eligible studies identified subsequent to the preparation of this protocol will be reviewed and included if they meet the eligibility criteria.

### Data extraction

Based on our search strategy a single reviewer will review the titles, abstracts and keywords of every record retrieved. Full articles will be retrieved for further assessment on the eligibility of the study for inclusion in the overview. Two reviewers will then independently select the studies for inclusion in the review from the list of potentially eligible studies. Details from eligible studies (methodological and nutritional) will be extracted independently by two reviewers using a pre-designed data extraction form. The data extraction form will include the following items:

#### General information (for both methodological and nutritional studies)

*Name of study, country where study was conducted, year of publication, journal, language of publication, contact address of corresponding author*.

#### Methodological information

*Was the method employed Bayesian or classical, can the method be implemented in standard statistical software or not, can the method deal with differential measurement error or not, can the method deal with correlated errors or not, can the method be used for continuous and categorical exposures or only a one type of exposure, does the method produce precise and valid estimates in a variety of settings (e.g. case-control or prospective cohort)*.

#### Dietary information

*Instruments for assessing dietary intake employed, reference instrument employed, was the dietary assessment instrument self-administered (e.g. computer) or not, if not self-administered how was it administered (e.g. interviewer), the timing of the administration; has the instrument been validated for the population it has been used on*.

The fundamental information relevant to each dietary assessment method is outlined in Table 1.

**Table 1:** Information to be extracted for each dietary assessment method

Dietary assessment method	Relevant information extracted
Food frequency (FFQ) questionnaire	• Number of food items
Non-weighed diet diary	• Number of days covered
Weighed records	• Number of days covered
24 hour recall	• Single or multiple recall • If multiple 24 hour recalls used what was the number of recalls? • Time interval between recalls
Biochemical marker	• Name of the biochemical marker?

#### Calibration information

The type of calibration study and the most relevant information to extract for each type of study is described briefly, in Table 2.

**Table 2:** Type of calibration study with typical design and information to be extracted

Type of calibration study	Typical design	Relevant information to extract
Reliability study	• Employs a comparison of several instruments to estimate dietary intake one of which may be a "gold-standard" • Uses a sub-sample of subjects from the main study or an external sample	• Number of instruments used • Number of subjects • Were biochemical markers used? • Was the sub-sample from main study or an external sample?
Validation study	• Employs a "gold-standard" compared with another instrument used to estimate dietary intake • Uses a sub-sample of subjects from the main study or an external sample	• Number of instruments used • Number of subjects • What was the "gold-standard" (biochemical marker/other instrument)? • Was the sub-sample from main study or an external sample?
Reproducibility study	• Employs replicate measurements for an instrument used to estimate dietary intake • Uses a sub-sample of subjects from the main study or an external sample	• Instrument used • Number of subjects • Number of repeat measures used, • Spacing of repeat measures, • Were biochemical markers used? • Was the sub-sample from main study or an external sample?

#### Measurement error information

*How measurement error was assessed, the assumptions of the method used to assess measurement error, the size of the correction factor obtained*.

Where the data extracted differ between assessors, the discrepancy will be resolved by consensus and when necessary, additional information will be sought from the authors of the studies. Where differences in opinion still exist, a third party will be consulted.

### Quality Assessment

The general quality of the methods used to estimate dietary intake will be assessed separately from the data extraction process using a series of questions devised by a member of the research team with expertise in nutrition, which will draw on information of existing scoring systems such as those described by Margetts et al. [17], and Friedenreich et al. [18] questions outlined in "Section II Method of Dietary Assessment" from a checklist devised by Nelson et al. [19], and a review of issues in the assessment of dietary intake, particularly for fruit and vegetables, by the International Agency for Research on Cancer [20]. Items of methodological quality will also be reported, including main study design details and the calibration study details. In addition, the assumptions of the methods used to correct for measurement error, and their relative advantages and limitations will be discussed.

### Timeframe for analyses

The aim of this review is to a) collate information on possible methods to correct for measurement error and b) present the magnitude of correction factors for different nutrients obtained from different study designs. The collection of correction factors for different nutrients can serve two purposes: 1) they can be used directly to calibrate results from different dietary assessment methods in multi-centre investigations, whenever calibration studies are not performed; 2) they can be used as prior evidence in Bayesian analyses of calibration studies

We have identified studies, either completed or ongoing, that are potentially eligible for inclusion in the both parts of the review. Data extraction should be completed by the end of the second quarter of 2012 and the preliminary analyses will be conducted in the third quarter of 2012.

## Discussion

Although it is not possible at this present stage to anticipate the methodological and nutritional results from this project we expect to have the following knowledge available by the final quarter of 2012:

1. To be able to adequately describe robust methodological approaches to the design of a calibration study in order to account for the effects of measurement error in a biobank

2. To be able to make the details of the different methodological approaches to correct for measurement error more accessible to a non-statistical audience.

3. Describe how to apply the correction factors to studies that aim to assess the association between diet and disease with data collected from multiple sites.

4. Ultimately, develop an inventory of the typical correction factors for various nutrients that are considered in individual epidemiological studies.

From the experiences in the present protocol we will provide recommendations on how these methods could be applied to prospective pooling of biobanks taking into account issues specific to these types of studies (such as G × E interactions). A final step is to provide these data through the internet via the main P^3^G website in order to disseminate our findings to researchers with similar interests.

## Competing interests

The authors declare that they have no competing interests.

## Authors' contributions

All authors participated in the design of the study. DB drafted the manuscript and JL, LFM and CM made critical revision of the manuscript for important intellectual content. All authors contributed to, read, and approved the final manuscript.

## Pre-publication history

The pre-publication history for this paper can be accessed here:

http://www.biomedcentral.com/1471-2288/11/135/prepub
